# Lost and Found: Misdiagnosis of AIDS-Related Bone Marrow Suppression As Neutropenic Fever and Benign Ethnic Neutropenia in a Patient With Congenital HIV

**DOI:** 10.7759/cureus.68632

**Published:** 2024-09-04

**Authors:** Ivy Huynh, Dillon M Woody, Mohammad A Ahmed-Khan, Victoria Garofalo, Tierney Grisolano, Quinn Willer

**Affiliations:** 1 Internal Medicine, Danbury Hospital, Danbury, USA; 2 Behavioral Health, Jackson Memorial Hospital, Miami, USA; 3 Internal Medicine, University of Vermont, Burlington, USA; 4 Internal Medicine, Yale School of Medicine, Danbury Hospital, Danbury, USA; 5 Medicine, American University of the Caribbean, Cupecoy, SXM; 6 Internal Medicine, American University of the Caribbean, Cupecoy, SXM

**Keywords:** neutropenia, secondary neutropenia, elevated liver associated enzymes, benign ethnic neutropenia, art non-adherence, anti-retroviral therapy (art), congenital hiv, pancytopenia, duffy-null phenotype, hiv aids

## Abstract

Neutropenia is a relatively uncommon but notable secondary effect of HIV infection. While the various hematopoietic effects of HIV and AIDS are well-described in the literature, high-quality evidence directly linking neutropenia with mortality in HIV-infected patients remains limited. The multifactorial etiology of neutropenia complicates its diagnosis, particularly when it occurs secondary to HIV. We present the case of a 35-year-old African American male with congenital HIV, who presented with severe neutropenia accompanied by a fever in the context of untreated HIV. The initial differential diagnosis was broad, including benign ethnic neutropenia (given the patient’s African American ethnicity), tuberculosis (given the potential for anti-tuberculosis therapy to cause neutropenia and its commonality as a co-infection in HIV patients), sepsis-related neutropenia, and AIDS-related bone marrow suppression. However, through further workup, it became apparent that HIV-related bone marrow suppression ultimately led to pancytopenia. This case highlights how HIV patient non-adherence to antiretroviral therapy (ART) and hematologic abnormalities complicate the diagnosis of hematopoietic abnormalities from HIV. It also discusses how vertical transmission and abrupt ART discontinuation create a new phenotype of HIV patients with delayed presentations of AIDS-related complications. This patient’s presentation also provides insight into the consequences of untreated HIV following the self-discontinuation of long-term HIV management therapy due to low healthcare literacy and loss of follow-up. The patient's clinical course, laboratory findings, imaging studies, and treatment outcomes are discussed, emphasizing the need for timely diagnosis and a multidisciplinary approach to care while exploring potential barriers to care in different social contexts.

## Introduction

Neutropenia in HIV-infected patients poses significant diagnostic and therapeutic challenges. In patients with congenital HIV who remain untreated for extended periods, complications such as neutropenia and opportunistic infections can be exacerbated. This case report details the presentation, diagnosis, and management of a patient with HIV-related bone marrow suppression who was initially misdiagnosed due to inconsistent adherence to antiretroviral therapy (ART), as self-reported by the patient. The differential diagnoses considered included benign ethnic neutropenia, neutropenic fever, and bone marrow suppression secondary to AIDS. Unlike the classic progression observed in congenital HIV patients, who may succumb to opportunistic infections such as candidiasis by adolescence or thrive with proper ART, this emerging phenotype presents unique challenges. Such individuals may experience severe disease manifestations due to lapses in treatment, complicating the traditional understanding of HIV's natural history.

## Case presentation

Clinical presentation and background

A 35-year-old male presented to the ED with a chief complaint of persistent cough, low-grade fever, mild dyspnea, and generalized weakness that had persisted for four months, starting in September 2023. The patient's medical history revealed congenital HIV, diagnosed at the age of five, with inconsistent adherence to ART before self-discontinuation at age 20. He also reported a history of heavy marijuana use but denied any significant cigarette smoking. Notably, the patient had recently relocated to the area and had not seen a physician in numerous years, resulting in a lack of continuity of care.

The patient’s cough and associated systemic symptoms did not resolve despite outpatient treatment with azithromycin and amoxicillin/clavulanic acid. Due to the chronicity of his symptoms, the patient was referred to the ED, where he was subsequently hospitalized from January 4th to January 17th for further evaluation and management.

Diagnostic workup and hospital course

Upon admission to the ED, the patient had a temperature of 37.7°C (100.0°F) and the remainder of his vital signs were unremarkable. Initial laboratory investigations revealed pancytopenia, with a WBC count of 1.1 x 10^3^/μL, an absolute neutrophil count (ANC) of 0.28 x 10^3/μL, platelets of 75 x 10^3/μL, and hemoglobin of 11.3 g/dL (Table 1).

**Table 1:** Serial complete blood count showing severe neutropenia and recovery following antiretroviral therapy. This table outlines the patient's complete blood counts at different stages during hospitalization and follow-up. The critical neutropenia (absolute neutrophil count of 0.28 x 10^3/μL) and other hematologic abnormalities improved after the initiation of antiretroviral therapy. μL: Cells per microliter; g/dL: Grams per deciliter.

Parameter	January 4, 2024	February 16, 2024	Reference Range
Neutrophil Absolute (x 10^3^/μL)	0.28 (Critical)	0.67	1.5-8.0
WBC (x 10^3/μL)	1.1	2.7	4.0-11.0
Platelets (x 10^3/μL)	70	201	150-400
Hemoglobin (g/dL)	11.6	12.0	13.5-17.5

The patient was then admitted to the medical ward due to concerns of neutropenia in the setting of fever with immunocompromise and possible pulmonary infection. The Infectious Diseases team was consulted for the management of untreated congenital HIV complicated by pneumonia. Infectious and serologic evaluations were performed (Table 2).

**Table 2:** Immunologic profile and HIV-related laboratory findings. This table shows the patient’s immunologic parameters, including CD4+ and CD8+ counts and ratios, across initial presentation and follow-up. The profound immunosuppression (CD4+ count of 2 cells/μL) is indicative of advanced AIDS. CD: Cluster of Differentiation; %: Percentage; /μL: Total number of specific cells per microliter of blood; CD3+: Cluster of Differentiation 3 Positive (T Lymphocytes); CD8+: Cluster of Differentiation 8 Positive (Cytotoxic T Cells); CD4+: Cluster of Differentiation 4 Positive (Helper T Cells); CD45+: Cluster of Differentiation 45 Positive (Leukocytes); CD3+/CD8+ Ratio: Ratio of CD3 Positive T Cells to CD8 Positive T Cells; CD3+/CD4+ Ratio: Ratio of CD3 Positive T Cells to CD4 Positive T Cells; CD4/CD8 Ratio: Ratio of CD4 Positive Helper T Cells to CD8 Positive Cytotoxic T Cells.

Parameter	January 5, 2024	February 16, 2024	Reference Range
CD3 + Percent (%)	61	73.9	50-75
CD3 + Absolute (x 10^3^/μL)	120	562	690-2540
CD3+/CD8+ Percent (%)	49.9	57.3	20-40
CD8+ Absolute (x 10^3^/μL)	98	436	180-1170
CD3+/CD4+ (%)	1.0	10.9	30 - 60
CD4+ Absolute (x 10^3^/μL)	2	83	500 - 1500
CD45 + Absolute (x 10^3^/μL)	197	761	800 - 2400
CD4/CD8 Ratio	0.02	0.19	0.9 - 1.9

Blood cultures, including Acid-fast bacilli (AFB), were unrevealing. A complete blood count (CBC) and peripheral smear were obtained demonstrating normochromic/normocytic anemia, no evidence of immune hemolysis or intraerythrocytic organisms, severe leukopenia, absolute neutropenia, rare reactive lymphocytes, monocytes and neutrophils, with no leukemic blasts, and thrombocytopenia with normal platelet morphology.

Given the patient's symptoms and HIV status, pulmonary tuberculosis (TB) was considered in the differential diagnosis. Initial testing included AFB smears, which were negative. To further evaluate for TB, Gene-Xpert MTB/RIF testing and liquid culture were conducted. Both tests were negative for *Mycobacterium tuberculosis*. The negative results from the liquid culture, the gold standard for TB diagnosis, ruled out pulmonary TB, allowing us to focus on other causes of the patient's symptoms and hematologic abnormalities.

**Figure 1:**
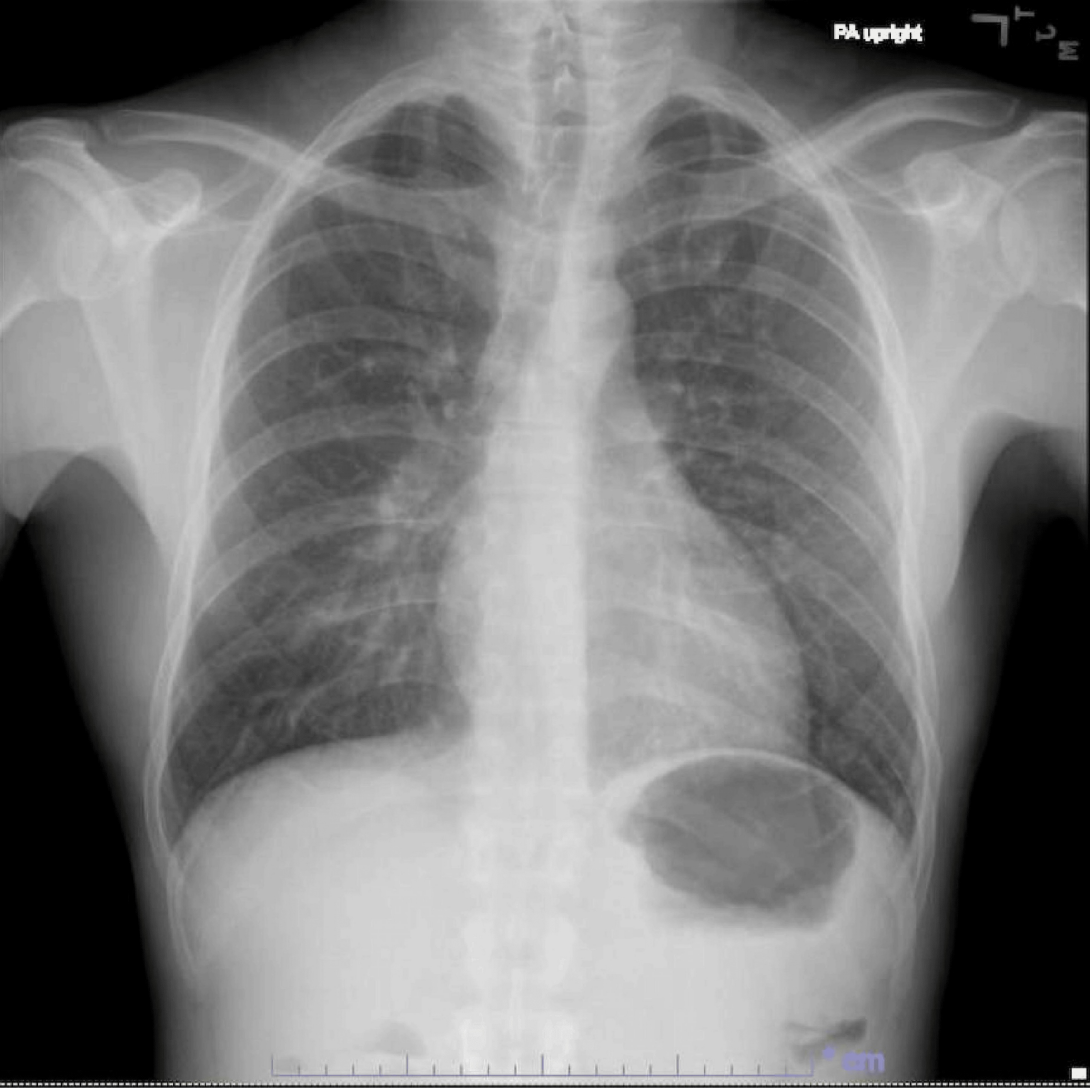
Chest X-ray demonstrating mild bronchial wall thickening without significant consolidation. The chest X-ray shows mild bronchial wall thickening with no evidence of consolidation, pleural effusion, or pneumothorax. The findings are suggestive of an early or mild infectious process, consistent with the patient’s clinical presentation of chronic cough and immunosuppression in the context of untreated HIV.

**Figure 2:**
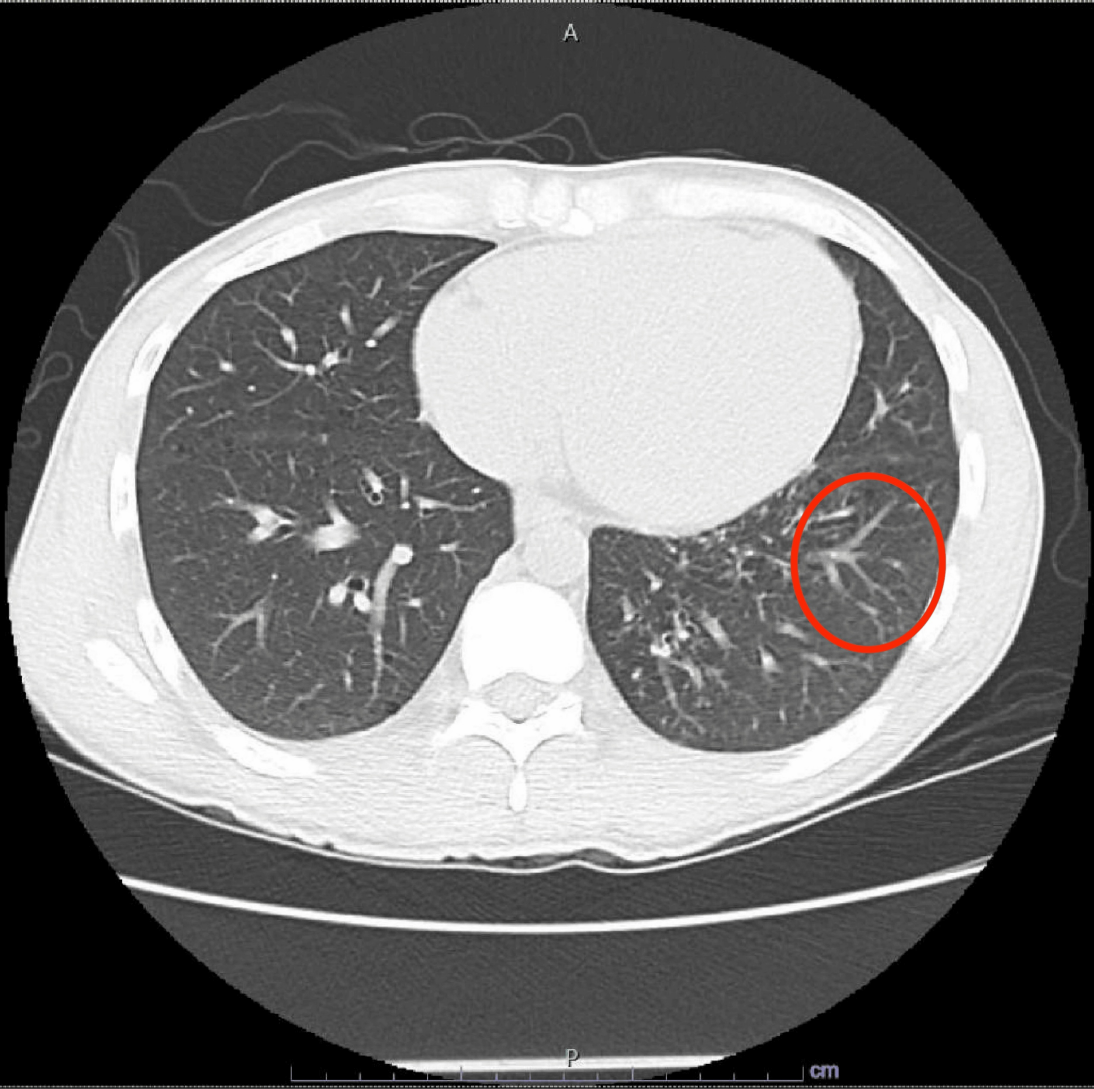
Axial non-contrast chest CT demonstrating a tree-in-bud pattern in the lower lobes. This image depicts the subtle tree-in-bud pattern with centrilobular nodules and branching opacities that give the appearance of budding tree branches, which may be indicative of early pneumonitis. The circled area highlights this finding in the left lung base.

Serologic tests were negative for *Toxoplasma gondii*, Parvovirus B19, and Cryptococcus. Cytomegalovirus (CMV) testing was negative for IgM antibodies but positive for IgG antibodies. Cryptococcal antigen from blood was negative. QuantiFERON results were indeterminate. HIV genotyping showed no significant resistance mutations, with an HIV PCR of 947,000 copies/mL. His absolute CD4+ count was 2/uL. A bone marrow biopsy revealed 'no evidence of *Mycobacterium avium* complex (MAC) infection' but showed 'scant maturing trilineage hematopoiesis with atypia and moderate reticulin fibrosis.' A chest X-ray revealed 'mild bronchial wall thickening, with no evidence of consolidation, pleural effusion, or pneumothorax' (Figure 1). An axial non-contrast chest CT demonstrated 'a subtle tree-in-bud pattern in the left lung base, which could represent early changes of a diffuse pneumonitis. Lungs demonstrate very small microcysts predominantly in the lung bases' (Figure 2). A contrast-enhanced CT of the chest, abdomen, and pelvis showed 'no significant lymphadenopathy or other acute findings.'

On admission, liver function tests (LFTs), including total bilirubin, alkaline phosphatase, and hepatic enzymes (aspartate aminotransferase (AST) and alanine aminotransferase (ALT)), were elevated and closely monitored throughout the hospitalization (Table 3). A hepatitis panel was ordered early in the admission and was negative for hepatitis A, B, C, and D. Alkaline phosphatase was initially elevated at 128 IU/L but trended down to within normal limits by January 12th, while AST and ALT levels also gradually improved during the hospital course.

**Table 3:** Trends in liver enzymes over the course of hospitalization. This table displays the patient’s liver enzyme levels from admission to follow-up. Elevated ALT and AST levels were observed initially and gradually normalized after antiretroviral therapy initiation, indicating resolving hepatic inflammation. Alk Phos: Alkaline Phosphatase; ALT: Alanine Transaminase; AST: Aspartate Aminotransferase; mg/dL: Milligrams per Deciliter; IU/L: International Units per Liter.

Date	Bilirubin Total (mg/dL)	Alk Phos (IU/L)	ALT (U/L)	AST (U/L)	Reference Range
January 4, 2024	0.4	128	161	317	Bilirubin: 0.1-1.2 mg/dL
					Alk Phos: 40-120 IU/L
					ALT: 7-56 U/L
					AST: 10-40 U/L
January 7, 2024	0.4	122	155	288	
January 8, 2024	0.4	124	165	332	
January 9, 2024	0.4	117	167	324	
January 10, 2024	0.4	104	156	280	
January 11, 2024	0.3	98	142	260	
January 12, 2024	0.4	96	135	225	
February 16, 2024	0.4	104	14	14	

Management and therapeutic interventions

During the admission, Biktarvy (bictegravir/emtricitabine/tenofovir Alafenamide), a fixed-dose combination antiretroviral medication, was initiated. Inpatient management included the broad-spectrum respiratory antibiotic therapy with high-dose IV trimethoprim/sulfamethoxazole (TMP-SMX) for pneumocystis pneumonia (PCP). Following negative* Pneumocystis jirovecii* PCR and silver screen results, the patient was transitioned to oral TMP-SMX for PCP prophylaxis.

At discharge, outpatient chemoprophylaxis with TMP-SMX and azithromycin was continued, along with Biktarvy for ART. Follow-up with infectious disease and hematology/oncology specialists was arranged to ensure a multidisciplinary approach to managing the patient’s complex case.

Given the patient’s challenging social circumstances, including insurance issues, medication costs, frequent relocations, low health literacy, and social stigma, a comprehensive discharge plan was prioritized. A pre-discharge meeting with the social work team connected the patient with state, city, and community-based support services to enhance adherence to care and aid in improving overall outcomes.

Follow-up and outcomes

At a follow-up visit in February, the patient showed improvement. He continued taking Biktarvy and antibiotics without adverse effects, with his chronic cough and associated symptoms, such as low-grade fever and fatigue, gradually improving. He supplemented his diet with nutritional shakes and reported no new complaints. Blood work demonstrated improvements in WBC count, ANC, and platelet count, along with minor gains in RBC count, hemoglobin, and hematocrit (Table 1). CD4+ count and liver enzymes also showed progress (Tables 2-3).

The follow-up plan included repeating the HIV PCR, CD4 count, and CBC with differential in two weeks, along with a follow-up QuantiFERON test after cellular reconstitution. The patient was advised to see a primary care physician for immunizations and to continue follow-ups with hematology/oncology for final bone marrow biopsy results and with infectious disease in 4-6 weeks.

## Discussion

This case represents an interplay of severe neutropenia and elevated liver enzymes in a patient with advanced HIV/AIDS, in the setting of ART non-adherence. The following discussion explores these key aspects, emphasizing the diagnostic challenges and broader implications for patient care and public health.

The patient’s clinical course was marked by chronic symptoms, including a persistent cough, low-grade fever, mild dyspnea, and generalized weakness, which failed to resolve despite outpatient treatment. His medical history revealed congenital HIV diagnosed at age 5, with inconsistent ART adherence leading to severe immunosuppression. An absolute CD4+ count of 2/μL indicated advanced HIV and AIDS, necessitating a multidisciplinary approach to care [1,2]. The patient’s pancytopenia reflected significant disruption of bone marrow function by HIV [1,2].

Vertical transmission of HIV and subsequent non-adherence with ART give rise to a unique phenotype of HIV patients. These individuals often face severe immunosuppression, higher viral loads, and increased susceptibility to opportunistic infections [3,4]. The clinical presentation of this subset of patients includes persistent viremia, rapid progression to AIDS-defining illnesses, and complex social and medical challenges [3,5]. Sudden discontinuation of ART, as seen in this patient, leads to significant disease progression and complications, highlighting the need for continuous and comprehensive care [3,4,5]. Managing such cases requires a multidisciplinary approach to address both medical and social challenges, as was done in the care of this patient, including ensuring access to treatment, continuity of care, and support services for the patient’s disease process [3,4].

Hematologic abnormalities in HIV

HIV-related hematologic abnormalities, such as anemia, thrombocytopenia, and neutropenia, are well-documented phenomena [1,2,6]. Pancytopenia, as seen in this patient, involves the reduction of all three blood cell lineages, consistent with severe bone marrow suppression [1] [2]. HIV can directly infect progenitor cells in the bone marrow and cause dysregulation of hematopoiesis, leading to cytopenias [1,7,8]. Additionally, chronic immune activation and opportunistic infections can further exacerbate these abnormalities [9,10].

The patient's bone marrow biopsy revealed moderate reticulin fibrosis, scant maturing trilineage hematopoiesis, and atypia. Reticulin fibrosis, often seen in HIV-infected individuals, indicates chronic inflammatory states due to persistent immune activation [2,8,11]. Scant maturing trilineage hematopoiesis suggests impaired production and maturation of blood cells, attributable to direct HIV infection of hematopoietic progenitor cells and the effects of inflammatory mediators [2,7,11]. Atypia in bone marrow cells may result from direct HIV infection, leading to abnormal cell morphology and function [2,8,12]. These findings collectively support the diagnosis of HIV-related bone marrow suppression, manifesting as pancytopenia and severe immunosuppression [1,2,7].

Neutropenia in HIV and diagnostic challenges

Neutropenia in HIV-infected patients poses significant diagnostic and therapeutic challenges. It can result from direct viral infection of hematopoietic stem cells, inflammatory mediators, and alterations in the bone marrow microenvironment [1,2]. Chronic HIV infection can lead to bone marrow suppression and resultant cytopenias due to direct infection of marrow cells, HIV-related immune activation, and opportunistic infections [1,7,8].

In the context of HIV, diagnosing neutropenia requires careful evaluation. Initially, the differential diagnosis may include benign ethnic neutropenia and even sepsis, particularly when there is inconsistent information regarding ART adherence [7,8,9]. The patient’s African American ethnicity initially raised the possibility of benign ethnic neutropenia, a condition commonly seen in individuals of African descent and characterized by lower baseline neutrophil counts without an increased risk of infection [9,10]. However, distinguishing this benign condition from pathologic neutropenia due to HIV is crucial to avoid unnecessary treatment and intervention [7,8].

The patient's presentation was further complicated by his history of inconsistent ART adherence, which added to the diagnostic complexity. Severe neutropenia, as observed in this patient with an ANC of 0.28 x 10^3^/μL, is more indicative of bone marrow suppression related to advanced HIV and AIDS rather than benign ethnic neutropenia [1,6,7]. This distinction was further supported by the patient’s bone marrow biopsy findings. These findings, coupled with the clinical presentation and history of ART non-adherence, confirmed HIV-related neutropenia [1,2,8]. This severe neutropenia, along with pancytopenia, emphasizes the impact of uncontrolled HIV on hematopoiesis and the critical need for accurate diagnosis and appropriate management in HIV-infected patients with hematologic abnormalities [1,2,4].

Liver enzyme abnormalities

Elevated liver enzymes are common in HIV-infected individuals and can result from ART toxicity, co-infections with hepatitis viruses, and direct effects of HIV on hepatocytes [4]. The patient’s liver enzyme assays showed elevated ALT and AST, which improved with treatment. This underscores the importance of monitoring hepatic function and addressing liver-related complications in HIV management [4]. Although ART-induced hepatotoxicity is a known cause of drug-induced liver injury (DILI) in HIV patients, especially with older antiretroviral drugs [4], this is unlikely in our patient due to the long discontinuation of ART. Additionally, HIV can directly infect liver cells, contributing to liver inflammation and injury [4]. The improvement in the patient's elevated liver enzymes following ART initiation, along with negative hepatitis serologies and the absence of recent drug exposure, suggests that the elevation was likely due to the direct effect of HIV on hepatocytes [4].

Public health implications

This case illustrates the critical importance of early HIV detection and continuous care. Adherence to ART is vital for managing HIV infection and preventing the development of drug-resistant strains [4,5]. Socioeconomic factors, healthcare access, and patient education play pivotal roles in ensuring adherence [4,5,10]. Addressing these barriers through patient-centered care and community support is essential for improving outcomes [4,5]. Patients with HIV, particularly those with a history of ART non-adherence, require comprehensive care that includes regular monitoring, patient education, and support services to address barriers to adherence [4,5].

Non-adherence to ART among patients with congenital HIV has been associated with poorer clinical outcomes, including higher viral loads, increased susceptibility to opportunistic infections, and rapid progression to AIDS-defining illnesses [3,4,5]. It has also been demonstrated that ART non-adherence leads to increased healthcare utilization (HCU) and costs, with non-adherent patients requiring more frequent hospitalizations and incurring higher overall healthcare expenses [4,5,13].

A critical issue in the management of HIV patients, particularly those with a history of non-adherence, is the risk of developing ART resistance. Intermittent or complete discontinuation of ART can lead to unchecked viral replication and the selection of drug-resistant strains [3,5]. This complicates future treatment options and increases the likelihood of treatment failure and further immunosuppression [5]. However, in this patient, the favorable response to ART during and following hospitalization indicates that resistance was likely not a significant factor. Continuous adherence to ART remains essential to prevent resistance and maintain long-term health in HIV-infected individuals [5].

## Conclusions

This case of a 35-year-old African American male with congenital HIV highlights the severe consequences of ART non-adherence, leading to pancytopenia, neutropenia, and elevated liver enzymes. The diagnostic complexity, particularly in differentiating between benign ethnic neutropenia, sepsis-related neutropenia, and advanced AIDS-related bone marrow suppression, emphasizes the intricate challenges of managing late-stage HIV. This case underscores the critical need for clinical vigilance and timely intervention, as untreated HIV can quickly progress to life-threatening complications. The findings reinforce the importance of sustained, multidisciplinary care in preventing such adverse outcomes.

Furthermore, this case sheds light on a vulnerable and increasingly prevalent patient population: individuals with congenital HIV who, due to socioeconomic barriers, experience significant lapses in care, resulting in a distinct phenotype marked by heightened disease severity. Effectively managing these patients requires more than medical intervention; it necessitates a holistic approach that integrates social support, patient education, and a nuanced understanding of the factors driving non-adherence. By advocating for integrative care models that prioritize equitable access and personalized follow-up, this report not only provides valuable insights into the evolving landscape of HIV management but also emphasizes the broader need for a healthcare system that is adaptive, inclusive, and compassionate in addressing the needs of underserved populations. Through these efforts, we can enhance patient outcomes and improve the quality of care delivered in similar complex cases.
